# Peroxisome Proliferator-Activated Receptor and Vitamin D Receptor Signaling Pathways in Cancer Cells

**DOI:** 10.3390/cancers5041261

**Published:** 2013-10-21

**Authors:** Satoru Matsuda, Yasuko Kitagishi

**Affiliations:** Department of Food Science and Nutrition, Nara Women’s University, Kita-Uoya Nishimachi, Nara 630-8506, Japan

**Keywords:** PPAR, RXR, vitamin D receptor, cell signaling, transcription

## Abstract

Peroxisome proliferator-activated receptors (PPARs) are members of the superfamily of nuclear hormone receptors, which respond to specific ligands such as polyunsaturated fatty acids by altering gene expression. Three subtypes of this receptor have been discovered, each evolving to achieve different biological functions. Like other nuclear receptors, the transcriptional activity of PPARs is affected not only by ligand-stimulation, but also by cross-talk with other molecules. For example, both PPARs and the RXRs are ligand-activated transcription factors that coordinately regulate gene expression. In addition, PPARs and vitamin D receptor (VDR) signaling pathways regulate a multitude of genes that are of importance for cellular functions including cell proliferation and cell differentiation. Interaction of the PPARs and VDR signaling pathways has been shown at the level of molecular cross-regulation of their transcription factor. A variety of ligands influencing the PPARs and VDR signaling pathways have been shown to reveal chemopreventive potential by mediating tumor suppressive activities in human cancers. Use of these compounds may represent a potential novel strategy to prevent cancers. This review summarizes the roles of the PPARs and the VDR in pathogenesis and progression of cancer.

## 1. Introduction

Nuclear receptor family is divided into two subfamilies. The first group includes the estrogen, androgen, progesterone and mineralocorticoid receptors [1], and the second group includes vitamin D receptor (VDR), the thyroid receptor (TR), retinoic acid receptor (RAR), retinoid X receptor (RXR), and peroxisome proliferator—activated receptors (PPARs) [2]. The second group of receptors can form heterodimers with each other, and can function through interacting with appropriate ligands at genetic level [1,2]. In particular, the PPARs and/or like the VDR represent a major research target for the understanding and treatment of many diseases [3]. After activation through a ligand, the PPARs and VDR form a heterodimer with the RXR and induce antitumor effects in a variety of human carcinomas [4]. Therefore, these pathways may play an important role in cancer treatment and prevention. Furthermore, modulating PPAR signaling pathways would represent a potential novel strategy for inhibiting carcinogenesis and progression. This review paper will focus on the evidence of the functions of PPARs and VDR in cancer.

## 2. Expression and Characteristics of PPARs and Vitamin D Receptor

**Figure 1:**
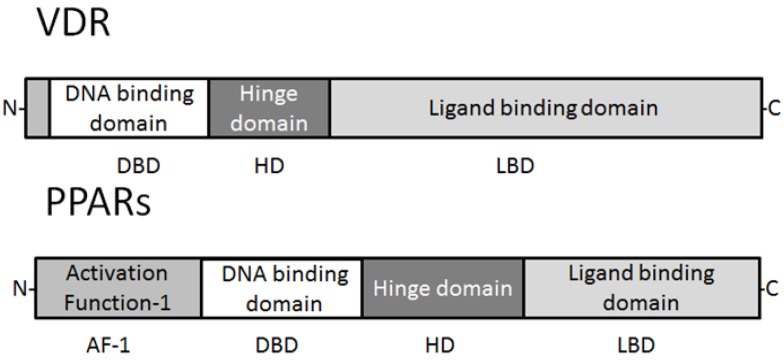
Schematic structure of VDR and PPAR protein. The predicted consensual important domain structures for VDR and PPAR are depicted, which are common in some species. AF-1 = activation function-1, DBD = DNA binding domain, HD = hinge domain linking DBD and LBD, LBD = C-terminal ligand binding domain. Note that the sizes of protein are modified for clarity.

**Figure 2:**
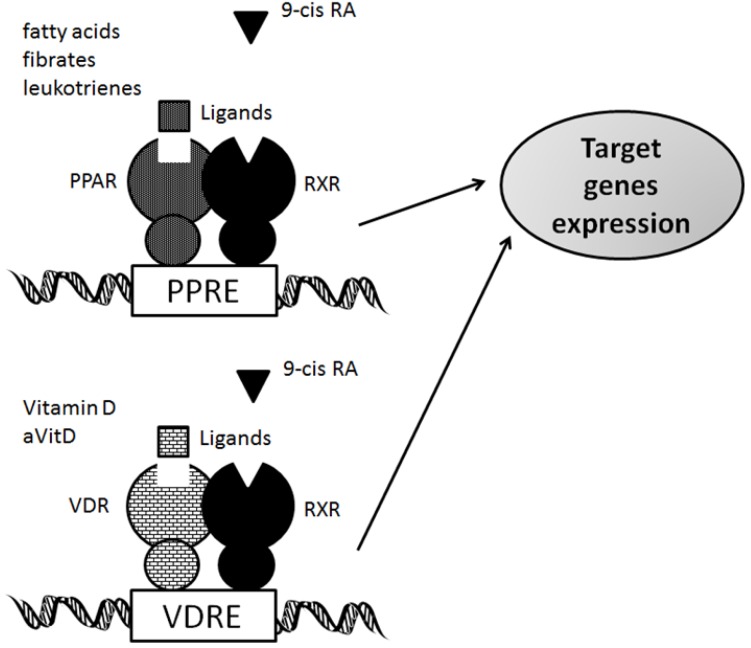
Schematic depiction of the model of mechanism of VDR and PPARs action. Similar to other nuclear hormone receptors, VDR and PPARs act as a ligand activated transcription factor. Both VDR and PPARs in response to their ligand binding, hetero-dimerize with RXR, and bind VDRE and PPRE DNA sequences in the promoters of target genes, respectively. Note that some critical molecules have been omitted for clarity.

PPARs are ligand-activated transcription factors that are involved in the genetic regulation of mammalian metabolism, including fatty acid oxidation, transport and metabolism mediating proteins through the formation of a DNA binding heterodimer complex [5,6,7]. These receptors have also been shown to be implicated in cellular proliferation, differentiation, tumor promotion, apoptosis and immune reaction/inflammation. Three genetically and functionally distinct PPAR isoforms, PPARs (PPARα, PPARβ/δ, and PPARγ), have been described. PPARα is expressed at high levels in tissues that catabolize fatty acids [8], as in the adult liver, heart, kidney, large intestine and skeletal muscle. PPARβ/δ mRNA is ubiquitously distributed with a higher expression in digestive tract and placenta [9]. PPARγ is mostly expressed in the adipose tissue [10] and immune system, and is an important regulator of their differentiation and metabolism. All distinct PPARs subtypes exhibit distinct patterns of tissue distribution and share a high degree of structural homology with other members of the superfamily, particularly in the DNA-binding domain and ligand-binding domain [5,7,11] (Figure 1). Each isotype is a product of a separate gene. Retinoic X receptor (RXR) is a functional partner of PPAR. RXRα and PPARγ function potently in metabolic diseases, and are both important targets for anti-diabetic drugs. Coactivation of RXRα and PPARγ is believed to synergize their effects on glucose and lipid metabolism [12]. The transcriptional regulation by PPARs requires heterodimerization with the retinoid X receptor (RXR) (Figure 2). PPARs bind to a variety of PPAR response elements (PPREs) present in the promoter regions of the responsive genes [13]. Thus, selective action of PPARs *in vivo* results from the interplay at a time point of each of the cofactors available. The RXRs are able to influence the transcription of a wide variety of genes, because they can activate gene transcription by binding to specific sites on DNA as homodimers and/or as the heterodimers with other related nuclear receptors including the PPARs, VDR, and TR, so forth [14,15,16]. A variety of compounds have been identified as PPARs ligands. Among the synthetic ligands, fibrates and thiazolidinediones are PPARα and PPARγ agonists, respectively [17]. A PPARα specific ligand, 8S-HETE, and a PPARγ specific ligand, PGJ, 15-deoxy-Δ^12,14^-prostaglandin J2, and a peroxisome proliferator, clofibrate, all are able to induce expression of both PPARα and PPARγ [18,19,20]. Subsequent work has led to the identification of various PPAR ligands that include eicosanoids, hypolipidemic agents, and antidiabetic drugs [21,22]. PPARγ is also activated by prostaglandins and leukotrienes [23]. Besides natural ligands such as polyunsaturated fatty acids (including linoleic acid, linolenic acid and arachidonic acid), a large number of synthetic PPAR ligands have been identified. Clinically used drugs like the thiazolidinediones (troglitazone and pioglitazone), a class of insulinsensitizing agents and the fibrates (bezafibrate and clofibrate), which are used as hypolipidaemic drugs, are also binding to the PPARs [24]. In the presence of ligands, conformational changes of the ligand binding domain result in the recruitment of co-activator proteins or release of co-repressor proteins, and following association of a protein complex that enhances transcription activity of the target genes [25,26].

The nutritional forms of vitamin D had been identified as 1,25(OH)2D3 (calcitriol), an active form of vitamin D (aVitD) [27]. The majority of the aVitD effect is thought to be carried out by VDR localized in the nucleus. The ligand of VDR was thus discovered before the cloning of the receptor. VDR is a high-affinity hormone receptor and a nonpermissive partner of RXR [28]. Ligand-bound VDR can regulate gene expression via various molecular mechanisms, including direct activation where VDR-RXR most effectively binds DR-3 (direct repeat with 3-bp spacer) response element [29]. The function of VDR is essential for promoting calcium absorption in the gut and maintaining adequate serum calcium and phosphate concentrations to enable normal mineralization of bone [30]. It is also required for bone growth and bone remodeling by osteoblasts and osteoclasts. VDR is expressed in most tissues in the human body, and vitamin D plays an important role in decreasing the risk of many chronic illnesses, cancers, autoimmune diseases, infectious diseases, and cardiovascular disease [31]. Vitamin D2 (ergocalciferol) has been shown to contribute to the vitamin D status in humans, and metabolized in a similar fashion as vitamin D3. Vitamin D3 can be synthesized in the skin when ultraviolet B (UVB) penetrates the skin [32]. In various cell types, including normal and cancer cells, the effects of aVitD and VDR mediated genomic pathways include the regulation of cell growth and differentiation. VDRE have been reported in the proximal promoter of a number of vitamin D-responding genes including the human vitamin D 24-hydroxylase (CYP24) [33,34]. The inactivation of vitamin D metabolites is carried out by the CYP24 that is a key enzyme in 24-hydroxylation.

## 3. Functional Interplay of Vitamin D Receptor with PPARs

Both PPARs and VDR could form heterodimers with the retinoid-X receptor (RXR). Both the VDR and PPAR compete for their predominant heterodimerisation partner, RXR, complex transcriptional regulation of target genes might be expected [35,36]. VDR associates with vitamin D and forms heterodimers with RXR and exerts its activity through binding to vitamin D response elements (VDREs) of target genes. In adipocyte, vitamin D and VDR inhibit both PPARγ activity and adipogenesis [35,37]. There is a potent VDRE in human PPAR promoter [38]. So, PPAR is a primary aVitD responding gene and that VDR and PPAR signaling pathways are interconnected at the level of cross-regulation of their respective transcription factor mRNA levels. This cross-talk may involve a competition for the same heterodimerisation partner, RXR and the presence of VDREs and peroxisome proliferator response elements (PPREs). Both PPAR ligands and vitamin D analogs have been shown to be implicated in tumor progression and cellular differentiation. After activation through a ligand binding, the conformation of PPARs and VDR is altered and stabilized, resulting in the creation of a binding fissure and recruitment of transcriptional coactivators. The provided link between the PPARs and the VDR is bidirectional with either side being able to influence the other’s activity. It has been shown that PPARγ binds to VDR and inhibits vitamin D mediated transactivation [39]. The cross-talk is also important for the ability of gene expression and regulates a multitude of genes that are of importance for various cellular functions including cell proliferation, cell differentiation, immune responses and apoptosis. The signaling pathways of the VDR and the PPARs are interconnected in a large number of cancer cell lines [40,41]. However, the complete mechanisms of this cross-talk between the VDR and PPAR signaling pathways are not yet known. Further investigations are required to evaluate the physiological and pathophysiological relevance of this cross-talk. If so, activation of PPAR signaling pathways by aVitD or other vitamin D ligands may open new perspectives for treatment or prevention of cancer cells.

## 4. PPARs and Vitamin D Receptor in Cancer

**Figure 3:**
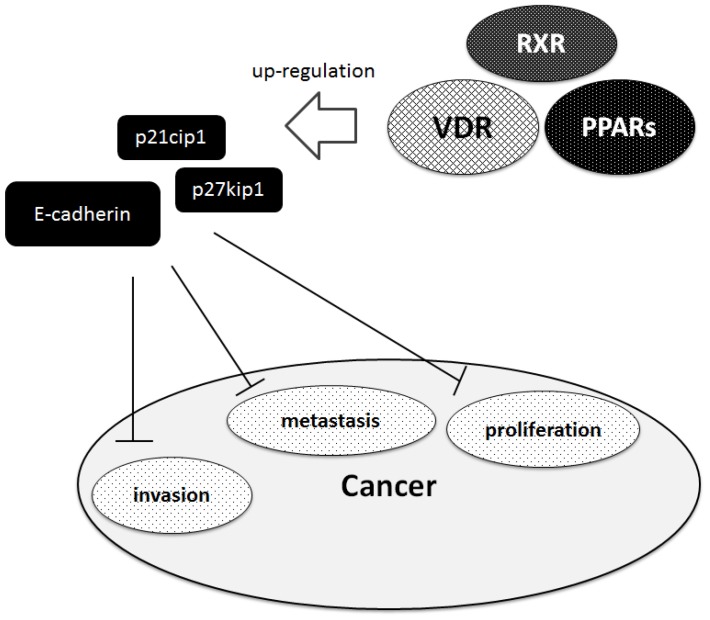
Implication of VDR, PPARs, and RXR in inhibition of cancer. VDR, PPARs, and RXR may be involved in inhibition of several aspect including proliferation, metastasis and invasion in cancer. Hammerheads mean inhibition. Note that some critical pathways have been omitted for clarity.

Generally, non‑steroidal nuclear receptors play a major role in cancer development. Antiproliferative effects of PPARs ligands could be demonstrated in different cell lines. Thiazolidinedione, a PPAR ligand, currently used to treat diabetes, inhibits the proliferation of cancer cells [42,43]. In addition, PPAR activation by corresponding ligands (ex. fenofibrate) decreases the metastatic potential via down-regulation of Akt signaling [44]. These antiproliferative effects are mediated by cell-cycle arrest through a PPAR dependent pathway. Therefore modulating PPAR signaling pathways represents a potential novel strategy for inhibiting carcinogenesis and its progression. Vitamin D also elicits antiproliferative effects in a variety of cancer cell types including cell lines derived from prostate. The anticancer mechanisms include induction of cell cycle arrest, promotion of differentiation, inhibition of proliferation and angiogenesis, as well as inhibition of invasive and migratory potential of cancer cells. The aVitD exerts a significant inhibitory effect on the G1/S checkpoint of the cell cycle by upregulating the cyclin dependent kinase inhibitors such as p27kip1 and p21cip1 [45]. Beside the growth regulation of cells, aVitD also has an effect on tumor invasion, angiogenesis and metastastic behavior in various malignancies [46,47]. Vitamin D and AKT inhibitors synergistically inhibit prostate cancer growth through induction of cell cycle [48]. Mechanisms involve in the aVitD-induced inhibition of tumor invasion and metastasis include inhibition of serine proteinases and metalloproteinases as well as the up-regulation of E-cadherin [49]. The VDR gene is a target of epithelial to mesenchymal transition (EMT) promoters [50]. Considering a number of target genes of VDR and PPARs, these nuclear factors may modulate proliferation and differentiation of normal and cancer cells via various mechanisms (Figure 3). Ligands and other agents influencing the PPAR and VDR signaling pathways have been shown to reveal chemopreventive potential by mediating tumor suppressive activities in a variety of human cancers [41]. Further studies have to show if PPARs and VDR open new perspectives as agents inhibiting malignant potential of cancer. The loss of anti-proliferative responsiveness in cancer cells toward ligands for VDR, RXRs, and PPARs may require underlying epigenetic events [51]. Actually, function of VDR can be modulated epigenetically by histone acetylation, and it cooperates with other nuclear receptors which are influenced by histone acetyl transferases as well as histone deacetylases [52].

## 5. Perspectives

The signaling pathways of PPARs and VDR regulate a multitude of genes that are of importance for various cellular functions. The findings of this study may therefore open new perspectives for treatment and/or prevention of cancers. Clearly, more work is needed to develop a comprehensive understanding of the cellular and molecular mechanisms in regulating PPARγ expression by vitamin D. Further study of PPARs, RXRs, and VDR functions in cancer cells may indicate pathways that are common to critical carcinogenic processes, providing additional focus for research in important cancers. In parallel, defining more specific mode of action by identifying the endogenous co-activators and modulators of these transcription factors in animal models will help to build more efficient therapeutic strategy. Future studies using functional genomic approaches will be required to more clearly establish the complicated mechanisms by which PPARs and VDR exert their actions. The link between the PPAR and VDR signaling pathways may help guide molecular-based treatment strategies and allow the synthesis of new agents for cancer treatment. Further investigations also have to show the benefit of PPAR ligands and aVitD compared to conventional chemotherapeutic regimens. 

## 6. Conclusions

Interaction of the PPARs and VDR signaling pathways has been shown at the level of molecular cross-regulation of their transcription factor in pathogenesis and progression of cancer. The link between the PPARs and VDR signaling pathways may guide molecular-based treatment strategies and allow the creation of new tools for cancer treatment.

## Abbreviations

aVitD: active form of vitamin D: 1,25(OH)2D3
AP-1: activator protein-1
EMT: mesenchymal transition
mRNA: messenger RNA
PPAR: peroxisome proliferator-activated receptor
PPRE: PPAR response element
RA: retinoic acid
RARs: retinoic acid receptors
RE: response element
RXR: retinoid X receptor
TR: thyroid receptor
VDR: vitamin D receptor
VDREs: vitamin D response elements
